# Iron and Parasites

**DOI:** 10.1155/2015/291672

**Published:** 2015-05-20

**Authors:** Rossana Arroyo, Theresa Ochoa, Jung-Hsiang Tai, Mireya de la Garza

**Affiliations:** ^1^Departamento de Infectómica y Patogénesis Molecular, Centro de Investigación y de Estudios Avanzados del Instituto Politécnico Nacional (CINVESTAV-IPN), Avenida IPN 2508, 07360 Mexico, DF, Mexico; ^2^Instituto de Medicina Tropical Alexander von Humboldt, Universidad Peruana Cayetano Heredia, Lima 031, Peru; ^3^School of Public Health, University of Texas, Houston, TX 77030, USA; ^4^Division of Infectious Diseases, Institute of Biomedical Sciences (IBMS), Academia Sinica, Taipei 11529, Taiwan; ^5^Departamento de Biología Celular, Centro de Investigación y de Estudios Avanzados del Instituto Politécnico Nacional (CINVESTAV-IPN), Avenida IPN 2508, 07360 Mexico, DF, Mexico

In this special issue, we analyze the importance of iron in the host-parasite interplay. Iron is a transition element and the fourth most abundant element in the Earth's crust. Iron is vital for growth of nearly all living organisms, from prokaryotes to humans. Iron plays an important role in several cellular processes, such as respiration, photosynthesis, oxygen transport, and DNA synthesis. Iron is essential but it is not easily bioavailable; ferric iron solubility is low at physiological pH whereas ferrous iron, in aerobic environments, is highly toxic. Therefore, iron is normally bound to proteins and the whole body and cellular iron concentrations have to be regulated in all organisms.

Some iron-containing and iron-binding proteins are intracellular such as the oxygen-carrier hemoglobin, the iron-storing protein ferritin, and numerous enzymes. Others are extracellular, mainly transferrin (Tf) and lactoferrin (Lf). Tf and Lf are able to capture up to two Fe^3+^ atoms per molecule, maintaining iron in a soluble and stable oxidation state in fluids and avoiding the generation of toxic free radicals derived from Fe^2+^ through the Fenton reaction. Free radicals are deleterious to most macromolecules. Tf and Lf maintain the free iron concentration too low to sustain the parasites growth. Tf is the iron transporter that allows cellular iron uptake; it is mainly found in serum and lymph. Lf is secreted into mucosae and by the secondary granules of neutrophils, to chelate the Fe^3+^ and avoid its availability for parasites.

Therefore, during infection, there is a constant battle between the host and the invader for iron, in which the invader attempts to have access to host iron and the host arranges complex iron-withholding mechanisms to frustrate the iron stealing. Virtually, all iron-containing proteins in eukaryotes can be used as iron sources by iron-seeking parasites; for that, several elaborate strategies have been developed by parasites to obtain host iron. Thus, capture and uptake of host iron by parasites are considered as virulence determinants.

Little information regarding iron acquisition in free-living amoebae has been reported. In the research article “Iron-Binding Protein Degradation by Cysteine Proteases of* Naegleria fowleri*,” M. Martínez-Castillo et al. report the cleaving of human hololactoferrin, hemoglobin, and holotransferrin by this parasite.* N. fowleri* causes primary amoebic meningoencephalitis. During the invasion, the microorganism interacts with different tissues such as olfactory neuroepithelium and olfactory bulbs that contain iron-binding proteins. The results show that this protozoan has several cysteine-secreted proteases that cleave iron-binding proteins. Using this strategy,* N. fowleri* could obtain iron from the host in the invaded tissues.

G. Ortíz-Estrada et al. address the issue about the possible way in which the human enteric parasite* Entamoeba histolytica* could have access to bovine lactoferrin, a protein present in the milk mainly consumed by babies and infants fed with formula. In their research article “Binding and Endocytosis of Bovine Hololactoferrin by the Parasite* Entamoeba histolytica*,” the authors compare virulent trophozoites recently isolated from hamster liver abscesses with nonvirulent trophozoites maintained for more than 30 years in* in vitro* cultures, regarding their interaction with bovine iron-charged Lf (B-holo-Lf). Interestingly, although both amoeba variants are able to use B-holo-Lf as an iron source and endocytosed this glycoprotein through clathrin-coated vesicles, the acquisition of iron, binding parameters, and number of protein-binding sites per amoeba are different. In addition, the virulent amoebae also endocytosed B-holo-Lf through a cholesterol-dependent mechanism; thus the B-holo-Lf endocytosis is more efficient in virulent amoebae.

In the minireview article “Strategies of Intracellular Pathogens for Obtaining Iron from the Environment,” N. Leon-Sicairos et al. focus on how intracellular pathogens use multiple approaches to obtain nutritional iron from the intracellular environment, in order to use this element for replication. They explore the current knowledge about the process that occurs during infection by intracellular pathogens, where the iron is required by both the host cell and the pathogen that inhabits it. Intracellular microorganisms are destroyed by the host tissues through processes that usually involve phagocytosis and lysosomal disruption. However, some intracellular pathogens are capable of avoiding destruction by growing inside macrophages and other cells. Additionally, the implications of these mechanisms for iron acquisition in the host-pathogen relationship are discussed.

African trypanosomosis is caused by the parasitic protozoan* Trypanosoma brucei*. This is a chronic and debilitating disease suffered mainly by people of developing countries. In the review “Iron-Homeostasis and* Trypanosoma brucei* Associated Immunopathogenicity Development: A Battle/Quest for Iron,” B. Stijlemans et al. analyze the different strategies that lead to a host immune response that results in iron deprivation, consisting in an iron modulation of the host myeloid phagocytic system that affects trypanosomosis-associated anemia development.

The review article “*Trichomonas vaginalis* Cysteine Proteinases: Iron Response in Gene Expression and Proteolytic Activity” by R. Arroyo et al. focuses on the iron response of* Trichomonas vaginalis* on gene family products as the cysteine proteinases (CPs) involved in virulence properties. In particular, it examines the effect of iron in gene expression regulation and function of cathepsin L-like and asparaginyl endopeptidase-like CPs as virulence factors. Aspects regarding CPs genomic organization are addressed to offer possible explanations to the fact that only few members of this large gene family are expressed at the RNA and protein levels. Also offers possible ways used to control these particular proteolytic activities. Moreover, all known iron regulatory mechanisms of CPs at transcriptional, posttranscriptional, and posttranslational levels along with new insights into the possible epigenetic and miRNA processes in* T. vaginalis* are also summarized.

Finally, in the review article “Transferrin: Endocytosis and Cell Signaling in Parasitic Protozoa,” by M. Reyes-López et al., the authors describe the presence of specific receptors for Tf in protozoan parasites. The signal transduction initiated upon ligand binding at the parasite plasma membrane with the process in mammalian cells is compared, based on the large amount of information on the Tf endocytosis. Several signaling pathways participate in Tf trafficking, such as the insertion of membrane vesicles, and the signaling pathways mediated by the inositol-1,4,5-triphosphate and diacylglycerol, MAPK, or growth factors. Some components of these pathways also found in parasites are included, as well as the identification of signaling proteins, useful in the study of essential factors for the parasitic life and as potential targets for the development of chemotherapeutic approaches.

We hope that researchers enjoy the reading of this special issue related to parasites and one of the most important chemical elements, iron. Undoubtedly, the acquisition of host iron by a pathogen is a crucial step during the development of infection and is determinant in its outcome.

*Rossana Arroyo* *Theresa Ochoa* *Jung-Hsiang Tai* *Mireya de la Garza*

